# Suicide Ideation, Plans, and Attempts Among Military Veterans vs Nonveterans With Disability

**DOI:** 10.1001/jamanetworkopen.2023.37679

**Published:** 2023-10-13

**Authors:** Rebecca K. Blais, Zhigang Xie, Anne V. Kirby, Nicole M. Marlow

**Affiliations:** 1 Arizona State University, Tempe; 2 Department of Public Health, University of North Florida, Jacksonville; 3 Department of Occupational and Recreational Therapies, University of Utah, Salt Lake City; 4 Department of Health Services Research, Management and Policy, University of Florida, Gainesville

## Abstract

**Importance:**

People with disability are at heightened risk for suicide ideation, planning, and attempt, with risk growing as the number of disabling limitations increases. Military veterans have higher rates of suicide deaths and disability relative to nonveterans.

**Objective:**

To evaluate whether veteran status is associated with greater risk for suicide in those with disability.

**Design, Setting, and Participants:**

This survey study used cross-sectional self-reported data from US adults who participated in the 2015-2020 National Survey on Drug Use and Health. Data were weighted to represent the population. Data analysis was conducted from July to August 2022.

**Main Outcomes and Measures:**

Suicide ideation, planning, and attempt served as primary outcomes. Disability status (present or absent) and number of disabling limitations (1, 2, or ≥3) served as factors. Veteran status was determined based on self-report (veteran or nonveteran). Multivariable logistic regression examined suicide ideation, planning, and attempt as a function of veteran status and disability variables.

**Results:**

Participants included 231 099 US veterans and nonveterans, representing 236 551 727 US adults, of whom 20.03% (weighted n = 47 397 876) reported a disabling limitation, 8.92% were veterans (weighted n = 21 111 727; 16.0% aged 35-49 years; 91.0% men; 6.7% Hispanic; 10.9% non-Hispanic Black; and 78.4% non-Hispanic White) and 91.08% were nonveterans (weighted n = 215 440 000; 25.4% aged 35-49 years; 44.0% male; 16.5% Hispanic; 11.7% non-Hispanic Black; and 63.3% non-Hispanic White). Overall, 4.39% reported suicide ideation, planning, or attempt (weighted n = 10 401 065). Among those with no disability, veteran status was associated with higher risk of suicide planning (adjusted odds ratio [AOR], 1.71; 95% CI, 1.17-2.49). Among those with 1 or 2 disabling limitations, being a veteran was associated with a lower risk of suicide planning (AOR, 0.57; 95% CI, 0.34-0.95) and history of attempt (AOR, 0.46; 95% CI, 0.24-0.88).

**Conclusions and Relevance:**

In this study of how suicide risk differs as a function of disability and veteran status, risk for death by suicide was lower among veterans with disability relative to nonveterans with disability. Veteran status may mitigate risk for suicide given increased receipt of more disability-related care through the Department of Veterans Affairs. Further research would extend this line of inquiry by examining the cause and type of disability as well as perceptions of disability on self-worth. It is possible that physical wounds of war are protective because of the meaning and value of service to one’s country.

## Key Points

**Question:**

Is service in the US military associated with suicide risk among those with disability?

**Findings:**

In this survey study with self-reported cross-sectional data from 231 099 US adults, representing more than 236 million individuals, service in the military was associated with higher suicide risk among those without disability. However, among those with disability, military service was associated with lower suicide risk compared with nonveterans with disability.

**Meaning:**

These findings suggest that military service could be a protective factor against suicide among a subpopulation of US adults.

## Teaser

This survey study evaluates whether veteran status is associated with greater risk for suicide among those with disability.

## Introduction

Suicide is a leading public health concern. In 2020, there were nearly 46 000 suicide deaths with more than 1.2 million suicide attempts.^1^ Suicide was listed as the 12th leading cause of death, at a rate of 13.48 per 100 000 people, with men at greatest risk.^1^ Also in 2020, 4.9% of US adults reported suicide ideation, 1.3% suicide planning, and 0.5% attempting suicide.^2^ Suicide and self-directed injury have high costs to society that extend beyond the emotional toll of losing individuals to a preventable cause of death. Notably, suicide behaviors cost more than $1.3 trillion/y in lifetime medical and work-loss expenditures.^3^

Among those at heightened risk for suicide are people with disability. Indeed, a recent US population-based study demonstrated that the presence of any disability impacting function was associated with a greater likelihood of reporting suicide ideation, suicide planning, and attempting suicide, with risk increasing as the number of disabling limitations increased.^4^ Unfortunately, and as observed in the literature,^5^ subgroups of people with disability at most heightened risk for death by suicide are not well known. Within the disability population, veterans of US military service are overrepresented,^6^ likely due to exposure to injury and death during service. The most common physical disabilities among veterans include tinnitus, hearing loss, and motor deficits.^7^ Veterans are also overrepresented in the population of individuals who die by suicide. In 2020, 6146 veterans died by suicide with an unadjusted rate of 31.7 per 100 000 people.^8^ This is over twice the general US population suicide rate,^1^ with elevated risk among veterans consistently observed over a 19-year period.^1^ As of 2020, suicide was the 2nd leading cause of death among service members and veterans younger than 45 years, and the 13th leading cause among all veterans.^8^ In sum, veterans are at greater risk for having a disability and for experiencing heightened risk for suicide relative to nonveterans. However, little is known about how veteran status may moderate the effect of disability status on risk for suicide-related outcomes.

Although suicide risk in the context of disability has not been compared between veterans and nonveterans, critical within group differences have been observed among veterans. Veterans with disability (VWD) or physical health difficulties exhibited heightened suicide risk and were more likely to attempt suicide relative to veterans without disability.^9,10,11,12,13,14^ However, among VWD, those who received lower or no disability-related benefits were at higher suicide risk,^15,16^ and receipt of benefits mitigated risk for suicide.^17^ Although existing studies highlight the complexity of disability and risk for suicide within military samples, current evidence has not directly compared risk of suicide-related outcomes between veteran and nonveteran samples with and without disability. The current study examined whether suicide ideation, planning, and attempt among those people with disability (self-reported functional difficulty in physical, mental, or emotional domains) differed by veteran status.

## Method

### Study Sample

This survey study was conducted in July and August 2022, using self-report, cross-sectional data from the 2015-2020 National Survey on Drug Use and Health (NSDUH). The NSDUH is an annual survey of the noninstitutionalized US population aged 12 years and older. Excluded from the NSDUH are individuals with no fixed household address, active-duty personnel, and residents of institutional settings (eg, correctional facilities, nursing homes). Independent multistage area probability sampling was used across 50 states and Washington, DC. While the NSDUH is designed for self-reporting, field interview data collection manuals include accommodations for respondents with severe physical impairment (eg, blindness, deafness).^18,19,20,21^ The NSDUH had 315 661 participants between 2015 and 2020, of whom 241 675 were aged 18 years or older. Of those, 239 631 had information related to disability, and 238 490 had information related to suicide ideation, planning, or attempt. Of these, 238 360 had data on veteran status, and a final total of 231 099 had data on relevant covariates, representing 236 551 727 US adults (weighted). These individuals make up the current study (Figure). This study was deemed exempt from review by the University of Florida Institutional Review Board and is in compliance with guidelines from the American Association for Public Opinion Research (AAPOR). The data set is publicly available; informed consent was obtained by the NSDUH team.

**Figure.**
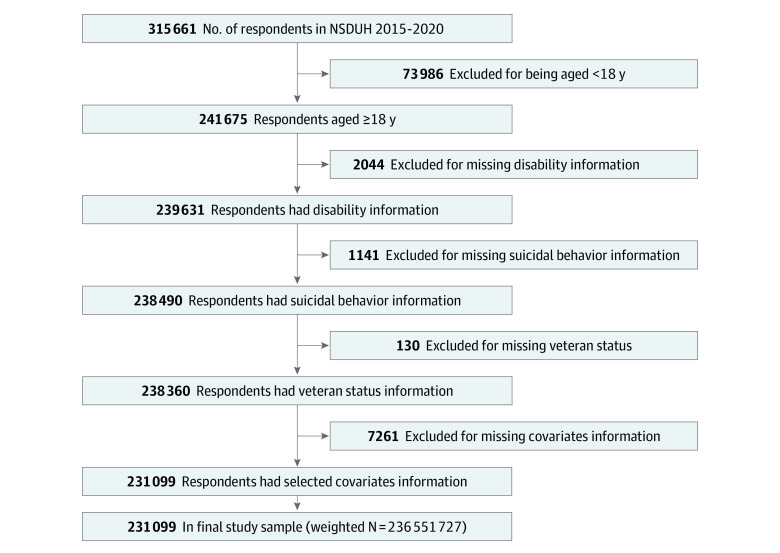
Study Cohort Selection Criteria and Process Flowchart of participant selection from the National Survey on Drug Use and Health (NDSUH) database.

### Measures

#### Risk for Suicide

Suicide ideation, planning, and attempt were assessed with 3 dichotomously scored questions: (1) “At any time in the past 12 months, did you seriously think about trying to kill yourself?” (if the response was yes, then questions 2 and 3 were asked); (2) “During the past 12 months, did you make any plans to kill yourself?”; and (3) “During the past 12 months, did you try to kill yourself?” Respondents were then categorized into 4 groups: (1) no risk for suicide (reference group, if no to question 1); (2) suicidal ideation only (if yes to question 1, and no to questions 2 and 3); (3) suicide planning without attempt (if yes to questions 1 and 2, and no to question 3); and (4) suicide attempt (if yes to questions 1 and 3, and either yes or no to question 2).

#### Disability

Disability status was assessed using 6 questions: (1) “Are you deaf or do you have serious difficulty hearing?”; (2) “Are you blind or do you have serious difficulty seeing, even when wearing glasses?”; (3) “Because of a physical, mental, or emotional condition, do you have serious difficulty concentrating, remembering, or making decisions?”; (4) “Do you have serious difficulty walking or climbing stairs?”; (5) “Do you have difficulty dressing or bathing?”; (6) “Because of a physical, mental, or emotional condition, do you have difficulty doing errands alone such as visiting a doctors’ office or shopping?” Responses were dichotomized to indicate presence of any functional disability with the following categorizations: (1) no functional disability (reference group, if no to all 6 questions) and (2) any functional disability (if yes to any of the 6 questions) and were coded by the number of disabling limitations, ranging from 0 to 3 or more.

#### Veteran Status

Veteran status was determined using the question: “Have you ever been in the United States Armed Forces?” Responses were dichotomized to indicate veteran status as (1) nonveteran (reference group, if no) and (2) veteran (if yes).

#### Covariates

Several sociodemographic characteristics, health status variables, and behavioral health information were included as covariates (Table 1). Urbanization was operationalized using the 2013 Rural-Urban Continuum Codes from the US Department of Agriculture.^22^

**Table 1.** Sociodemographic and Clinical Characteristics of 231 099 Respondents, Representing 236 551 727 US Adults, by Veteran Status

Characteristic	Weighted No.	Weighted % (95% CI)		*P* value
		Nonveteran	Veteran	
Suicide-related outcome				
None	226 150 662	95.5 (95.4-95.7)	96.2 (95.7-96.7)	.002
Suicidal ideation only	7 250 065	3.1 (3.0-3.2)	2.8 (2.3-3.2)	
Suicide planning without attempt	1 892 256	0.8 (0.8-0.8)	0.8 (0.6-0.9)	
Suicide attempt	1 258 744	0.6 (0.5-0.6)	0.3 (0.2-0.4)	
Disability status				
No disabling limitations	189 153 851	80.9 (80.6-81.3)	70.1 (68.8-71.3)	<.001
1 Limitation	28 668 870	11.6 (11.4-11.9)	17.1 (16.1-18.0)	
2 Limitations	10 307 907	4.2 (4.0-4.3)	6.5 (5.9-7.1)	
≥3 Limitations	8 421 099	3.3 (3.1-3.4)	6.4 (5.8-7.1)	
Age group, y				
18-25	32 389 043	14.8 (14.6-15.0)	2.2 (2.0-2.4)	<.001
26-34	37 632 422	16.8 (16.5-17.0)	7.0 (6.5-7.5)	
35-49	58 188 169	25.4 (25.1-25.7)	16.0 (15.3-16.7)	
50-64	59 629 883	25.1 (24.8-25.5)	26.0 (24.7-27.4)	
≥65	48 712 209	17.8 (17.5-18.2)	48.8 (47.3-50.4)	
Sex				
Male	114 071 243	44.0 (43.7-44.4)	91.0 (90.3-91.7)	<.001
Female	122 480 484	56.0 (55.6-56.3)	9.0 (8.3-9.7)	
Race and ethnicity^a^				
Hispanic	36 895 346	16.5 (16-16.9)	6.7 (6.2-7.3)	<.001
Non-Hispanic Asian	13 041 090	5.9 (5.7-6.2)	1.1 (0.9-1.4)	
Non-Hispanic Black	27 618 505	11.7 (11.4-12.1)	10.9 (10-11.8)	
Non-Hispanic American Indian or Alaska Native	1 255 177	0.5 (0.5-0.6)	0.4 (0.3-0.5)	
Non-Hispanic Native Hawaiian or Other Pacific Islander	806 711	0.4 (0.3-0.4)	0.2 (0.1-0.4)	
Non-Hispanic White	152 886 446	63.3 (62.7-63.9)	78.4 (77.4-79.5)	
Non-Hispanic ≥2 races	4 048 452	1.7 (1.6-1.7)	2.2 (1.9-2.5)	
Marital status				
Married	122 886 850	50.7 (50.2-51.2)	64.3 (63.1-65.6)	<.001
Not married	113 664 877	49.3 (48.8-49.8)	35.7 (34.4-36.9)	
Education				
Less than high school education	28 068 126	12.5 (12.2-12.7)	5.9 (5.2-6.5)	<.001
High school graduate	58 951 843	24.8 (24.5-25.1)	26.5 (25.2-27.8)	
Some college/associate degree	73 531 775	30.5 (30.1-30.8)	37.3 (36.0-38.5)	
College graduate	75 999 983	32.3 (31.8-32.8)	30.4 (29.4-31.4)	
Employment				
Employed	147 832 875	63.9 (63.5-64.2)	48.5 (47.1-49.8)	<.001
Unemployed	88 718 852	36.1 (35.8-36.5)	51.5 (50.2-52.9)	
Family income, $				
<20 000	37 355 287	16.4 (16.0-16.7)	9.9 (9.0-10.8)	<.001
20 000-49 999	68 314 946	28.7 (28.4-29.1)	30.3 (29.2-31.4)	
50 000-74 999	37 775 373	15.7 (15.5-15.9)	18.8 (17.8-19.8)	
≥75 000	93 106 121	39.2 (38.7-39.7)	41.0 (39.7-42.3)	
Insurance coverage				
Yes	213 458 643	89.6 (89.4-89.8)	96.5 (96.0-96.9)	<.001
No	23 093 084	10.4 (10.2-10.6)	3.5 (3.1-4)	
Urbanization				
Metropolitan	202 568 465	86.0 (85.5-86.4)	82.2 (81.2-83.3)	<.001
Not metropolitan	33983262	14.0 (13.6-14.5)	17.8 (16.7-18.8)	
Ever smoked				
Yes	144 010 155	59.4 (59.0-59.7)	76.4 (75.5-77.3)	<.001
No	92 541 572	40.6 (40.3-41.0)	23.6 (22.7-24.5)	
Alcohol dependence				
No	228 906 823	96.7 (96.6-96.8)	97.3 (97.0-97.7)	.006
Yes	7 644 904	3.3 (3.2-3.4)	2.7 (2.3-3.0)	
Illicit drug dependence				
No	231 366 279	97.7 (97.6-97.8)	98.9 (98.6-99.1)	<.001
Yes	5 185 448	2.3 (2.2-2.4)	1.1 (0.9-1.4)	
Health status				
Excellent	49 274 970	21.3 (21.1-21.6)	15.6 (14.8-16.4)	<.001
Very good	85 901 677	36.6 (36.2-37.0)	33.4 (32.2-34.7)	
Good	69 047 678	28.9 (28.5-29.2)	32.6 (31.2-33.9)	
Fair/poor	32 327 403	13.2 (12.9-13.5)	18.4 (17.5-19.3)	
No. of ED visits				
0	176 854 241	75.1 (74.7-75.4)	71.8 (70.6-73.0)	<.001
1	33 709 050	14.0 (13.8-14.3)	16.4 (15.6-17.2)	
2	16 769 146	7.1 (6.9-7.3)	7.3 (6.6-7.9)	
≥3	9 219 290	3.8 (3.7-4.0)	4.5 (3.9-5.1)	
No. of chronic conditions				
0	136 742 666	59.6 (59.2-60.0)	39.7 (38.7-40.7)	<.001
1	63 881 044	26.5 (26.2-26.8)	32.2 (31.1-33.4)	
≥2	35 928 016	13.9 (13.6-14.2)	28.1 (27.0-29.2)	
Depressive episode				
Yes	17 464 535	7.5 (7.4-7.7)	6.0 (5.5-6.5)	<.001
No	219 087 192	92.5 (92.3-92.6)	94.0 (93.5-94.5)	

Abbreviation: ED, emergency department.

^a^ Participants could self-report any combination of racial identities, including American Indian or Alaska Native, Asian, Black or African American, Guamanian or Chamorro, Samoan, Native Hawaiian, Other Pacific Islander, White, and Other. The National Survey on Drug Use and Health public use data combines this information with Hispanic origin to recode and create the 7-level race and ethnicity variable reported here. Those who identified as more than 1 race were categorized as 2 or more.

### Statistical Analysis

Analyses were conducted using SAS, version 9.4 (SAS Institute), which accommodated the complex NSDUH survey design and nonresponses by using PROC SURVEY procedures (incorporating person-level analysis survey weights, sampling unit clusters, and strata). Analyses utilized NSDUH’s weighted data to produce nationally representative estimates. Sample characteristics were computed using univariate statistics. Descriptives and selection of adjusted model covariates were computed using bivariate analyses. Covariates that had significant associations with veteran status, disability, and suicide-related outcomes were included in multivariable models.

Multivariable adjusted multinomial logistic regression with an interaction term for disability status × veteran status estimated adjusted odds ratios (AORs) and 95% CIs for suicide ideation, planning, and attempt by disability and veteran status. Sensitivity analyses were performed with multiple imputation by chained equations for all missing values to assess bias. A subgroup analysis was performed for individuals with suicide ideation, consistent with the ideation-to-action framework,^23,24,25,26^ which suggests risk for suicide is truly most problematic among those with suicidal thoughts. In these analyses, those reporting suicidal ideation only (but not planning or attempt) served as the reference group. Test-specific significance levels were assessed at α ≤ .05. Multicollinearity was assessed using the variance inflation factor but was not detected.

## Results

### Descriptive Statistics

The final sample included 231 099 participants, representing 236 551 727 US adults (weighted), of whom 20.03% (weighted n = 47 397 876) reported a disabling limitation. Of those with limitations, 60.5% reported 1 limitation, 21.7% reported 2 limitations, and 17.8% reported 3 or more limitations. Risk for suicide was reported by 4.39% (weighted n = 10 401 065), with 69.71% reporting suicide ideation only, 18.19% reporting suicide planning, and 12.10% reporting suicide attempt. Veterans represented 8.92% of the sample (n = 13 859; weighted n = 21 111 727; 16.0% aged 35-49 years; 91.0% men; 6.7% Hispanic; 10.9% non-Hispanic Black; and 78.4% non-Hispanic White), and 91.08% were nonveterans (n = 217 240; weighted n = 215 440 000; 25.4% aged 35-49 years; 44.0% male; 16.5% Hispanic; 11.7% non-Hispanic Black; and 63.3% non-Hispanic White). As shown in Table 1, the majority identified as non-Hispanic White, were married, aged 50 to 64 years, were female individuals, and did not report disability, suicide risk, or chronic condition. The majority were college graduates, employed, insured, lived in a metro area, and made $75 000/year or more. The majority did not report history of depression or alcohol or drug dependence, rated their health as “very good,” and did not report a recent visit to the emergency department. Most reported some exposure to cigarette use. Veterans more frequently reported disabling limitations, older age, being male, being non-Hispanic White or Hispanic, being married, varying levels of education and income, having insurance coverage, residing in an urban area, being smokers, poorer health, utilization of emergency services, and chronic health conditions, while they were less likely to report alcohol or drug dependence and history of a depressive episode (Table 1). All evaluated covariates had significant bivariate associations (eTables 1 and 2 in Supplement 1) and were included in adjusted analyses.

### Suicide Ideation, Planning, and Attempt and Disability Status

Among veterans only, and compared with having no disabling limitations, suicide ideation was 50%, 160%, and 127% more likely among VWD reporting 1, 2, and 3 or more disabling limitations, respectively; suicide planning was 114% more likely among VWD reporting 3 or more disabling limitations; and suicide attempt was 160% and 157% more likely among VWD reporting 1 and 3 or more disabling limitations, respectively (Table 2). Results from sensitivity analyses were also significant (eTable 3 in Supplement 1).

**Table 2.** Adjusted Multinomial Logistic Regression Model of Suicide-Related Outcomes by Disability and Veteran Status^a^

No. of disabling limitations by veteran status	Suicidal ideation only (vs no suicide-related outcome)			Suicide planning without attempt (vs no suicide-related outcome)			Suicide attempt (vs no suicide-related outcome)		
	AOR (95% CI)	*P* value	AME (95% CI)	AOR (95% CI)	*P* value	AME (95% CI)	AOR (95% CI)	*P* value	AME (95% CI)
Veteran									
No limitations^b^	1 [Reference]	NA	NA	1 [Reference]	NA	NA	1 [Reference]	NA	NA
1 Limitation	1.50 (1.01 to 2.23)^c^	.04	0.01 (0 to 0.03)	0.81 (0.45 to 1.46)	.48	0 (−0.01 to 0)	2.60 (1.46 to 4.66)^c^	.001	0.01 (0 to 0.01)
2 Limitations	2.60 (1.59 to 4.24)^c^	<.001	0.03 (0.01 to 0.06)	1.77 (0.73 to 4.29)	.21	0.01 (−0.01 to 0.02)	1.39 (0.64 to 2.99)	.40	0 (−0.01 to 0.01)
≥3 Limitations	2.27 (1.34 to 3.86)^c^	<.001	0.03 (0 to 0.06)	2.14 (1.13 to 4.07)^c^	.02	0.01 (0 to 0.02)	2.57 (1.13 to 5.82)^c^	.02	0 (0 to 0.01)
Nonveteran									
No limitations^b^	1 [Reference]	NA	NA	1 [Reference]	NA	NA	1 [Reference]	NA	NA
1 Limitation	2.02 (1.80 to 2.27)^c^	<.001	0.02 (0.02 to 0.03)	2.43 (2.04 to 2.90)^c^	<.001	0.01 (0 to 0.01)	2.13 (1.78 to 2.55)^c^	<.001	0 (0 to 0.01)
2 Limitations	2.32 (1.96 to 2.74)^c^	<.001	0.03 (0.02 to 0.03)	2.93 (2.39 to 3.61)^c^	<.001	0.01 (0.01 to 0.01)	2.93 (2.40 to 3.57)^c^	<.001	0.01 (0 to 0.01)
≥3 Limitations	2.65 (2.30 to 3.06)^c^	<.001	0.03 (0.02 to 0.04)	3.20 (2.28 to 4.49)^c^	<.001	0.01 (0 to 0.01)	3.68 (2.69 to 5.05)^c^	<.001	0.01 (0 to 0.01)
No limitations									
Nonveteran^b^	1 [Reference]	NA	NA	1 [Reference]	NA	NA	1 [Reference]	NA	NA
Veteran	1.12 (0.88 to 1.42)	.35	0 (−0.01 to 0)	1.71 (1.17 to 2.49)^c^	.006	0 (0 to 0)	0.98 (0.60 to 1.59)	.92	0 (0 to 0)
1 Limitation									
Nonveteran^b^	1 [Reference]	NA	NA	1 [Reference]	NA	NA	1 [Reference]	NA	NA
Veteran	0.83 (0.59 to 1.18)	.30	to 0.01 (−0.02 to 0.01)	0.57 (0.34 to 0.95)^c^	.03	0 (−0.01 to 0)	1.19 (0.70 to 2.04)	.52	0 (−0.01 to 0.01)
2 Limitations									
Nonveteran^b^	1 [Reference]	NA	NA	1 [Reference]	NA	NA	1 [Reference]	NA	NA
Veteran	1.25 (0.81 to 1.95)	.32	0 (−0.02 to 0.04)	1.03 (0.45 to 2.38)	.95	0 (−0.01 to 0.01)	0.46 (0.24 to 0.88)^c^	.02	0 (−0.01 to 0)
≥3 Limitations									
Nonveteran^b^	1 [Reference]	NA	NA	1 [Reference]	NA	NA	1 [Reference]	NA	NA
Veteran	0.96 (0.58 to 1.58)	.87	0 (−0.03 to 0.03)	1.14 (0.65 to 2.03)	.65	0 (−0.01 to 0.01)	0.68 (0.32 to 1.45)	.32	0 (−0.01 to 0.01)

Abbreviations: AME, average marginal effect; AOR, adjusted odds ratio; NA, not applicable.

^a^ Adjusted for age, sex, race and ethnicity, marital status, education, employment status, household income, insurance status, urbanization, smoking status, alcohol dependence, illicit drug dependence, health status, number of emergency department visits, number of comorbidities, depressive episode, and year of survey.

^b^ Reference group.

^c^ *P* ≤ .05.

In the subgroup analysis among those reporting ideation, VWD reporting 1 limitation had 51% lower odds of planning relative to those reporting no disabling limitations (Table 3). This result was also significant in sensitivity analyses (eTable 4 in Supplement 1).

**Table 3.** Adjusted Multinomial Logistic Regression Model of Suicide-Related Outcomes by Disability and Veteran Status Among Those With Suicidal Ideation^a^

No. of disabling limitations by veteran status	Suicide planning without attempt (vs suicidal ideation only)			Suicide attempt (vs suicidal ideation only)		
	AOR (95% CI)	*P* value	AME (95% CI)	AOR (95% CI)	*P* value	AME (95% CI)
Veteran						
No limitations^b^	1 [Reference]	NA	NA	1 [Reference]	NA	NA
1 Limitation	0.49 (0.24 to 0.97)^c^	.04	−0.11 (−0.22 to 0)	1.42 (0.68 to 2.97)	.35	0.04 (−0.06 to 0.14)
2 Limitations	0.70 (0.27 to 1.85)	.48	−0.06 (−0.21 to 0.10)	0.44 (0.18 to 1.07)	.07	−0.07 (−0.16 to 0.02)
≥3 Limitations	0.93 (0.44 to 1.94)	.84	−0.01(−0.15 to 0.13)	1.14 (0.43 to 3.00)	.80	0.02 (−0.09 to 0.13)
Nonveteran						
No limitations^b^	1 [Reference]	NA	NA	1 [Reference]	NA	NA
1 Limitation	1.29 (1.05 to 1.57)^c^	.01	0.04 (0.01 to 0.07)	1.09 (0.90 to 1.31)	.38	0.01 (−0.02 to 0.03)
2 Limitations	1.31 (1.04 to 1.64)^c^	.02	0.04 (0.01 to 0.08)	1.28 (1.04 to 1.58)^c^	.02	0.03 (0 to 0.06)
≥3 Limitations	1.30 (0.92 to 1.86)	.14	0.04 (−0.01 to 0.10)	1.36 (0.98 to 1.89)	.07	0.04 (−0.01 to 0.05)
No limitations						
Nonveteran^b^	1 [Reference]	NA	NA	1 [Reference]	NA	NA
Veteran	1.61(1.03 to 2.49)^c^	.04	0.08 (0 to 0.16)	0.90 (0.53 to 1.53)	.69	−0.02 (−0.08 to 0.05)
1 Limitation						
Nonveteran^b^	1 [Reference]	NA	NA	1 [Reference]	NA	NA
Veteran	0.61 (0.35 to 1.05)	.07	−0.07 (−0.15 to 0)	1.17 (0.63 to 2.18)	.62	0.02 (−0.08 to 0.11)
2 Limitations						
Nonveteran^b^	1 [Reference]	NA	NA	1 [Reference]	NA	NA
Veteran	0.87 (0.37 to 2.04)	.74	−0.02 (−0.16 to 0.12)	0.31 (0.14 to 0.66)^c^	.002	−0.11 (−0.18 to −0.04)
≥3 Limitations						
Nonveteran^b^	1 [Reference]	NA	NA	1 [Reference]	NA	NA
Veteran	1.14 (0.58 to 2.25)	.70	0.03 (−0.10 to 0.15)	0.75 (0.31 to 1.81)	.52	−0.03 (−0.14 to 0.07)

Abbreviations: AME, average marginal effect; AOR, adjusted odds ratio; NA, not applicable.

^a^ Adjusted for age, sex, race and ethnicity, marital status, education, employment status, household income, insurance status, urbanization, smoking status, alcohol dependence, illicit drug dependence, health status, number of emergency department visits, number of comorbidities, depressive episode, and year of survey.

^b^ Reference group.

^c^ *P* ≤ .05.

Among nonveterans, and compared with those with no disabling limitations, higher odds for suicide ideation, planning, and attempt were observed among those reporting 1, 2, and 3 or more disabling limitations, with risk for each increasing as the number of limitations increased (Table 2). For suicide ideation, risk increased by 102% to 165%; for suicide planning, risk increased by 143% to 220%; and for suicide attempt, risk increased by 113% to 268%. Results retained significance in sensitivity analyses (eTable 3 in Supplement 1).

In subgroup analyses among those reporting ideation, nonveterans reporting 1 or 2, but not 3 or more, disabling limitations were 29% to 31% more likely to report planning relative to those without disabling limitations (Table 3). However, nonveterans reporting 3 or more disabling limitations were more likely to report planning in sensitivity analyses, and the associations of 1 or 2 disabling limitations retained significance (eTable 4 in Supplement 1). Moreover, nonveterans reporting 2 disabling limitations were 28% more likely to report attempt relative to those with no disabling limitations (Table 3). This result was significant in sensitivity analyses (eTable 3 in Supplement 1). Finally, nonveterans reporting 3 or more disabilities were 102% more likely to report attempt in sensitivity analyses only (eTable 4 in Supplement 1).

### Suicide Ideation, Planning, and Attempt by Veteran and Disability Status

Among those reporting no disabling limitations, veterans were 71% more likely to report planning relative to nonveterans (AOR, 1.71; 95% CI, 1.17-2.49) (Table 2), but this was not significant in sensitivity analyses (eTable 3 in Supplement 1). However, among people reporting 1 disabling limitation, VWD had 43% lower odds of planning compared with nonveterans (AOR, 0.57; 95% CI, 0.34-0.95). Also, among people reporting 2 disabling limitations, VWD had 54% lower odds of attempt compared with nonveterans (AOR, 0.46; 95% CI, 0.24-0.88) (Table 2). These results remained significant in sensitivity analyses (eTable 3 in Supplement 1). No significant differences in risks for suicide were observed by veteran status among people reporting 3 or more disabling limitations.

### Suicide Planning and Attempt Among Those Reporting Suicide Ideation

In the subgroup analysis among those reporting ideation, veterans with no disabling limitations were 61% more likely than their nonveteran counterparts to report planning (Table 3). VWD with 2 disabling limitations were 69% less likely to report attempt relative to their nonveteran counterparts (Table 3). These results were both retained in sensitivity analyses (eTable 4 in Supplement 1). Lastly, veterans with 1 disabling limitation were more likely to report planning relative to their nonveteran counterparts, but this finding was only significant in sensitivity analyses (eTable 4 in Supplement 1).

## Discussion

The current study sought to broaden the literature on disability and suicide risk by examining how veteran status affected this association using a national sample of US individuals. The study builds on previous work using the NSDUH, which showed that risk for suicide was heightened among people with disability, with risk escalating as the number of disabling limitations increased.^4^ In the current study, results were not uniform across all levels of risk for suicide and disability status, but were uniform among veteran status: being a US veteran mitigated the association of disability status and risk for suicide, but in the absence of disability, veterans were at heightened suicide risk.

The observed buffering effect of veteran status among people with disability may be reflective of characteristics of disability-related care (DRC) offered through the Department of Veterans Affairs (VA). It is possible that VA services could act as a protective factor for suicide-related outcomes for VWD by improving access, quality of care, and understanding of their disability context. Furthermore, the VA states that suicide prevention is their “top clinical priority,”^27^ which is likely better reflected at all levels of care. Other individuals with disability often face a myriad of challenges to accessing health care,^28^ and health care professionals may be less aware of elevated suicide risk^29^ for the nonveteran disability population. This is speculative as the veteran group was not limited to those using VA health services, nor was assessment for receipt of DRC among veterans included in the NSDUH.

Of note, the literature is rich on the association of increased service utilization in the VA among VWD^30^ and the usefulness of adaptive interventions for veterans to improve quality of life, improve perceptions of disability,^26^ and provide access to adaptive sporting engagement^31^ as well as satisfaction with VA adaptive services.^32^ Thus, available evidence suggests that the VA provides a host of services to veterans to mitigate the challenges of living with disability, which could attenuate risk for suicide. Additionally, if the disability is the result of military service, these services are provided for free or at low cost, increasing accessibility compared with other people with disability who often lack affordable and accessible care.^33^ Furthermore, a recent study of veterans with service-connected disability showed that those who utilized mental health services through the VA were less likely to attempt suicide.^34^ This line of inquiry would be furthered by identifying mechanisms of the association of lower risk for suicide, disability status, and service in the US military, including receipt of DRC.

Although we cannot confirm that disability status was linked to military service, some of the conditions included in our assessment of disability are common limitations secondary to military service (eg, hearing loss).^7^ Research shows that greater acceptance of disability is associated with higher quality of life.^35^ It is possible that having a disability secondary to military service is protective as it is evidence of a sacrifice to one’s country. It had been argued that invisible wounds of war, such as posttraumatic stress disorder or major depressive disorder, are highly stigmatized conditions^36^ and connote weakness.^37^ Thus, more visible or physical wounds, such as physical disabilities, may be better tolerated by a population whose physical fitness is intricately linked with readiness and ability.^38^ The current investigation did not specifically compare mental disability with physical disability, so this interpretation should be considered with caution.

Although the current study identified an important moderator of the association of disability status and risk for suicide, future research is needed to better establish potential reasons for these associations. In addition to the possibilities noted previously (ie, DRC services, pride or acceptance of disability, lower stigma), interpersonal factors should be considered. Leading theories of suicide highlight the role of disjointed interpersonal function as a strong correlate of suicide risk.^24,39^ It is possible that disability status relates to risk for suicide through higher perceived burden to others, and other relationship difficulties.^40^ Given the infrastructure for veterans to receive care for disability through the VA, it is possible this increased contact facilitates better adjustment.

The current study adds to the body of literature on suicide planning in military samples. As noted in a recent meta-analysis on suicide in this population,^41^ relative to suicide ideation and attempt, suicide planning is far less studied. From a clinical perspective, suicide planning represents a more concerning level of risk for suicide relative to suicidal ideation. Thus, additional studies that explore factors before suicide attempt are critically needed to develop more intervention points to prevent an attempt. The NSDUH asked generally about a suicide plan, yet studies of suicide planning suggest various aspects to explore within this domain, such as the difference between identifying a specific method vs identifying a time and place.^42^ Thus, the current study builds on this literature base but also helps identify future areas of inquiry.

### Limitations

The current study has limitations that should be acknowledged when considering these findings. Disability status and count did not assess severity, so it is unclear how severity of disability relates to risk for suicide among veterans and nonveterans. Moreover, all assessments were based on self-report, and there is evidence of shame and stigma following suicide and self-directed violence,^43^ suggesting that more objective measures of suicide risk may be useful. Whereas we covaried for depression status, extant literature shows that psychiatric comorbidities, including posttraumatic stress disorder, exacerbate the challenges secondary to disabling limitations, suggesting additional research could examine how psychiatric status impacts these associations.^44^ Furthermore, due to smaller cell sizes after stratifying sample groups to veteran and nonveterans, we were unable to examine whether specific types of disability confer unique risks for suicide. Previous investigations on the association of suicide and disability using this data have shown meaningful differences between conditions,^4,45^ suggesting utility in looking at specific disabilities and their role in suicide. Additionally, data collection was cross-sectional, so we cannot distinguish the temporality of independent and outcome variables. All the findings should be only interpreted as associations; a systematic review of disability and risk for suicide noted the importance of examining these associations over time.^5^

## Conclusions

This study builds on the previous literature on risk for suicide and disability status by exploring veteran status as a moderator. Whereas most studies comparing outcomes among nonveterans and veterans demonstrate veterans to be at higher risk, our study highlights how prior military service may be protective against suicide when considering disability status.

## Notes

Supplement 1. **eTable 1.** Respondents’ Sociodemographic and Clinical Characteristics by Suicide-Related Outcomes (N = 231 099, Weighted N = 236 551 727) **eTable 2.** Respondents’ Sociodemographic and Clinical Characteristics by Disability Status (N = 231 099, Weighted N = 236 551 727) **eTable 3.** Multiple Imputation Sensitivity Analysis for Adjusted Multinomial Logistic Regression Model of Suicide-Related Outcomes by Disability and Veteran Status (N = 241 675) **eTable 4.** Multiple Imputation Sensitivity Analysis For Adjusted Multinomial Logistic Regression Models of Suicide-Related Outcomes Among Those With Suicidal Ideation

Supplement 2.
Data Sharing Statement
