# Supplementary material for: Suicide Ideation, Plans, and Attempts Among Military Veterans vs Nonveterans With Disability
Source: JAMA Netw Open. 2023 Oct 13;6(10):e2337679. doi: 10.1001/jamanetworkopen.2023.37679 (PMC10576218; doi:10.1001/jamanetworkopen.2023.37679)
Supplement: Supplement 1. — eTable 1. Respondents’ Sociodemographic and Clinical Characteristics by Suicide-Related Outcomes (N = 231 099, Weighted N = 236 551 727) eTable 2. Respondents’ Sociodemographic and Clinical Characteristics by Disability Status (N = 231 099, Weighted N = 236 551 727) eTable 3. Multiple Imputation Sensitivity Analysis for Adjusted Multinomial Logistic Regression Model of Suicide-Related Outcomes by Disability and Veteran Status (N = 241 675) eTable 4. Multiple Imputation Sensitivity Analysis For Adjusted Multinomial Logistic Regression Models of Suicide-Related Outcomes Among Those With Suicidal Ideation [file jamanetwopen-e2337679-s001.pdf]

Supplemental Online Content

Blais RK, Xie Z, Kirby AV, Marlow NM. Suicide ideation, plans, and attempts among military veterans vs nonveterans with disability. *JAMA Netw Open.* 2023;6(10):e2337679. doi:10.1001/jamanetworkopen.2023.37679

eTable 1. Respondents' Sociodemographic and Clinical Characteristics by Suicide-Related Outcomes (N = 231 099, Weighted N = 236 551 727)

eTable 2. Respondents' Sociodemographic and Clinical Characteristics by Disability Status (N = 231 099, Weighted N = 236 551 727)

eTable 3. Multiple Imputation Sensitivity Analysis for Adjusted Multinomial Logistic Regression Model of Suicide-Related Outcomes by Disability and Veteran Status (N = 241 675)

eTable 4. Multiple Imputation Sensitivity Analysis For Adjusted Multinomial Logistic Regression Models of Suicide-Related Outcomes Among Those With Suicidal Ideation

This supplemental material has been provided by the authors to give readers additional information about their work.

eTable 1. Respondents' Sociodemographic and Clinical Characteristics by Suicide-Related Outcomes (N = 231 099, Weighted N = 236 551 727)

Characteristic	Weighted No.	Weighted % (95% CI)				*P*
		**No suicide-related outcome**	**Suicidal ideation only**	**Suicide planning without attempt**	**Suicide attempt**	
Disability status						<.001
No limitations	189153851	81.2 (81.0-81.6)	55.4 (53.9-57.0)	44.5 (41.1-47.8)	44.5 (40.8-48.1)	
1 limitation	28668870	11.6 (11.3-11.9)	22.8 (21.5-24.2)	25.9 (23.0-28.7)	23.8 (21.5-26.1)	
2 limitations	10307907	4.0 (3.8-4.1)	11.9 (10.7-13.1)	15.9 (14.0-17.7)	16.3 (14.1-18.5)	
≥3 limitations	8421099	3.2 (3.1-3.4)	9.8 (8.7-10.9)	13.8 (11.6-16.1)	15.5 (12.5-18.4)	
Disability Status, by type						<.001
No limitations	189153851	81.2 (81.0-81.6)	55.4 (53.9-57.0)	44.5 (41.1-47.8)	44.5 (40.8-48.1)	
Hearing limitation, only	6538196	2.8 (2.7-3.0)	2.1 (1.6-2.6)	0.4 (0.1-0.8)	1.1 (0.5-1.6)	
Vision limitation, only	4205734	1.8 (1.7-1.9)	1.8 (1.5-2.2)	2.4 (1.3-3.5)	2.1 (1.4-2.8)	
Cognitive limitation, only	8083120	2.9 (2.8-3.0)	13.5 (12.3-14.7)	16.4 (14.6-18.2)	16.1 (13.9-18.3)	
Mobility limitation, only	7800438	3.3 (3.2-3.5)	2.3 (1.6-3.0)	2.8 (1.6-4.0)	1.4 (0.7-2.1)	
Complex activity limitation, only	2041382	0.8 (0.7-0.8)	3.1 (2.6-3.5)	3.9 (2.4-5.4)	3.2 (2.4-4.0)	
≥2 limitations	18729006	7.2 (7.0-7.4)	21.7 (20.4-23.1)	29.7 (26.7-32.7)	31.7 (28.4-35.0)	
Veteran status						0.002
Veteran	21111727	9.0 (8.8-9.2)	8.0 (7.0-9.1)	8.4 (6.7-10.2)	4.7 (3.2-6.2)	
Nonveteran	215440000	91.0 (90.8-91.2)	92.0 (90.9-93.0)	91.6 (89.8-93.3)	95.3 (93.8-96.8)	
Age group (years)						<.001
18-25	32389043	12.9 (12.7-13.0)	29.1 (27.9-30.3)	33.5 (31.2-35.9)	45.4 (42.2-48.7)	
26-34	37632422	15.7 (15.4-15.9)	22.2 (20.6-23.7)	20.2 (18.5-21.9)	17.2 (14.9-19.6)	
35-49	58188169	24.7 (24.4-25.0)	21.3 (20.1-22.6)	24.3 (21.6-27.1)	19.0 (15.8-22.2)	
50-64	59629883	25.6 (25.2-26.0)	17.5 (16.0-18.9)	16.9 (13.9-19.8)	12.9 (10.0-15.9)	
65+	48712209	21.1 (20.8-21.5)	9.9 (8.3-11.5)	5.1 (3.8-6.4)	5.4 (3.5-7.3)	
Sex						<.001
Male	114071243	48.3 (48.0-48.7)	46.7 (44.6-48.7)	46.0 (42.4-49.7)	40.0 (36.5-43.6)	
Female	122480484	51.7 (51.3-52.0)	53.3 (51.3-55.4)	54.0 (50.3-57.6)	60.0 (56.4-63.5)	
Race/ethnicity						<.001
Hispanic	36895346	15.7 (15.3-16.1)	13.7 (12.6-14.7)	13.8 (11.5-16.0)	16.6 (14.1-19.2)	
NH Asian	13041090	5.6 (5.4-5.8)	3.6 (3.0-4.2)	3.2 (2.2-4.1)	4.4 (3.0-5.8)	
NH black	27618505	11.8 (11.4-12.1)	9.4 (8.7-10.1)	9.2 (6.9-11.5)	14.0 (11.9-16.0)	
NH NAA	1255177	0.5 (0.5-0.6)	0.5 (0.3-0.7)	0.9 (0.5-1.3)	1.4 (0.4-2.3)	
NH NHOPI	806711	0.3 (0.3-0.4)	0.3 (0.1-0.4)	0.2 (0.1-0.3)	0.5 (0-1.3)	
NH white	152886446	64.5 (63.9-65.0)	69.7 (68.3-71.1)	68.9 (65.8-72.0)	59.5 (56.4-62.7)	

© 2023 Blais RK et al. *JAMA Network Open.*

NH 2+ races	4048452	1.6 (1.6-1.7)	2.8 (2.2-3.3)	4.0 (3.1-4.8)	3.6 (2.6-4.7)	
Marital status						<.001
Married	122886850	52.9 (52.4-53.4)	32.9 (31.3-34.5)	28.3 (25.3-31.3)	18.3 (15.4-21.1)	
Not married	113664877	47.1 (46.6-47.5)	67.1 (65.5-68.7)	71.7 (68.7-74.7)	81.7 (78.9-84.6)	
Education						<.001
Not high school graduate	28068126	11.9 (11.6-12.2)	10.2 (9.0-11.4)	9.7 (7.4-12.0)	18.4 (15.6-21.2)	
High school graduate	58951843	24.9 (24.6-25.2)	23.4 (22.0-24.8)	27.1 (24.4-29.7)	33.0 (29.8-36.1)	
Some college/associate degree	75999983	30.7 (30.3-31.0)	40.2 (38.5-41.8)	42.2 (39.4-45.0)	36.5 (33.0-40.0)	
College graduate	236551727	32.5 (32.0-33.0)	26.2 (24.6-27.8)	21.0 (18.5-23.5)	12.1 (9.9-14.4)	
Employment						<.001
Employed	147832875	62.6 (62.2-63.0)	62.4 (60.7-64.2)	59.2 (56.5-61.9)	50.1 (46.8-53.5)	
Unemployed	88718852	37.4 (37.0-37.8)	37.6 (35.8-39.3)	40.8 (38.1-43.5)	49.9 (46.5-53.2)	
Family income						<.001
<$ 20,000	37355287	15.4 (15.1-15.7)	22.3 (21.0-23.5)	28.0 (24.5-31.6)	32.8 (29.2-36.3)	
$20,000-49,999	68314946	28.7 (28.3-29.1)	32.4 (30.5-34.2)	31.0 (27.9-34.1)	34.3 (31.1-37.5)	
$50,000-74,999	37775373	16.0 (15.8-16.3)	14.9 (13.6-16.1)	15.3 (12.9-17.6)	11.9 (10.1-13.7)	
$75,000+	93106121	39.9 (39.3-40.4)	30.4 (28.8-32.1)	25.7 (23.0-28.4)	21.1 (18.2-23.9)	
Insurance coverage						<.001
Yes	213458643	90.4 (90.2-90.6)	87.2 (86.4-88.0)	85.8 (83.8-87.7)	83.5 (81.0-85.9)	
No	23093084	9.6 (9.4-9.8)	12.8 (12.0-13.6)	14.2 (12.3-16.2)	16.5 (14.1-19.0)	
Urbanization						<.001
Metro	202568465	85.6 (85.2-86.1)	86.2 (85.0-87.4)	83.9 (82.1-85.7)	84.0 (81.3-86.6)	
Not metro	33983262	14.4 (13.9-14.8)	13.8 (12.6-15.0)	16.1 (14.3-17.9)	16.0 (13.4-18.7)	
Ever Smoked						<.001
Yes	144010155	60.5 (60.1-60.8)	70.2 (68.8-71.5)	68.9 (65.8-71.9)	71.8 (68.7-74.8)	
No	92541572	39.5 (39.2-39.9)	29.8 (28.5-31.2)	31.1 (28.1-34.2)	28.2 (25.2-31.3)	
Alcohol Dependence						<.001
No	228906823	97.2 (97.0-97.3)	89.3 (88.4-90.2)	87.3 (85.6-88.9)	83.2 (80.7-85.7)	
Yes	7644904	2.8 (2.7-3.0)	10.7 (9.8-11.6)	12.7 (11.1-14.4)	16.8 (14.3-19.3)	
Illicit Drug Dependence						<.001
No	231366279	98.2 (98.2-989.3)	89.9 (88.7-91.1)	87.4 (85.5-89.4)	80.1 (77.3-82.9)	
Yes	5185448	1.8 (1.7-1.8)	10.1 (8.9-11.3)	12.6 (10.6-14.5)	19.9 (17.1-22.7)	
Health Status						<.001
Excellent	49274970	21.3 (21.0-21.5)	11.3 (10.4-12.3)	11.3 (9.7-12.9)	11.3 (9.7-12.9)	
Very good	85901677	36.6 (36.2-36.9)	32.2 (30.5-33.8)	29.7 (27.3-32.1)	26.4 (23.7-29.1)	
Good	69047678	29.0 (28.6-29.3)	34.8 (33.3-36.3)	31.7 (29.1-34.3)	33.3 (30.1-36.5)	
Fair/poor	32327403	13.2 (12.9-13.5)	21.7 (20.2-23.2)	27.3 (25.2-29.5)	29.0 (26.1-31.8)	
Number of ED visits						<.001
0	176854241	75.5 (75.1-75.8)	63.9 (62.2-65.7)	55.3 (52.4-58.2)	39.6 (35.7-43.4)	

© 2023 Blais RK et al. *JAMA Network Open.*

1	33709050	14.0 (13.8-14.3)	18.5 (17.0-20.0)	19.0 (16.5-21.4)	24.5 (21.2-27.7)	
2	16769146	6.9 (6.7-7.1)	10.4 (9.4-11.3)	12.3 (10.4-14.2)	14.1 (11.4-16.8)	
3+	9219290	3.6 (3.5-3.7)	7.2 (6.3-8.0)	13.4 (11.5-15.4)	21.9 (18.9-24.8)	
Number of chronic conditions						<.001
0	136742666	57.9 (57.6-58.3)	55.6 (54.1-57.1)	52.0 (48.9-55.1)	55.3 (51.9-58.7)	
1	63881044	27.0 (26.7-27.2)	27.2 (25.7-28.7)	27.2 (25.1-29.3)	30.3 (27.4-22.3)	
2+	35928016	15.1 (14.8-15.3)	17.2 (15.6-18.8)	20.8 (17.8-23.7)	14.4 (11.9-16.9)	
Depressive episode						<.001
Yes	17464535	5.3 (5.2-5.5)	47.1 (45.7-48.5)	67.6 (64.9-70.3)	54.5 (50.6-58.5)	
No	219087192	94.7 (94.5-94.8)	52.9 (51.5-54.3)	32.4 (29.7-35.1)	45.5 (41.5-49.4)	

Abbreviations: NH, non-Hispanic; ED, emergency department; CI, confidence interval; NAA, Native American/Alaska Native; NHOPI, Native Hawaiian/Other Pacific Islander

© 2023 Blais RK et al. *JAMA Network Open.*

eTable 2. Respondents' Sociodemographic and Clinical Characteristics by Disability Status (N = 231 099, Weighted N = 236 551 727)

Characteristic	**Weighted No.**	**Weighted % (95% CI)**				***p***
		**No limitations**	**1 limitation**	**2 limitations**	**≥3 limitations**	
Suicide-related outcome						<.001
None	226150662	97.1 (97.0-97.2)	91.5 (91.0-91.9)	86.7 (85.7-87.7)	86.2 (84.8-87.5)	
Suicidal ideation only	7250065	2.1 (2.0-2.2)	5.8 (5.4-6.2)	8.4 (7.6-9.2)	8.4 (7.4-9.5)	
Suicide planning, no attempt	1892256	0.4 (0.4-0.5)	1.7 (1.5-1.9)	2.9 (2.6-3.3)	3.1 (2.5-3.7)	
Suicide attempt	1258744	0.3 (0.3-0.3)	1.0 (0.9-1.2)	2.0 (1.7-2.3)	2.3 (1.8-2.8)	
Veteran status						<.001
Veteran	21111727	7.8 (7.6-8.1)	12.6 (11.8-13.3)	13.3 (12.1-14.4)	16.1 (14.4-17.8)	
Nonveteran	215440000	92.2 (91.9-92.4)	87.4 (86.7-88.2)	86.8 (85.6-87.9)	83.9 (82.2-85.6)	
Age group (years)						<.001
18-25	32389043	14.3 (14.1-14.5)	13.4 (13-13.9)	11.6 (11-12.1)	4.4 (3.9-4.9)	
26-34	37632422	17.4 (17.1-17.7)	11.1 (10.7-11.6)	9.9 (9.0-10.8)	5.5 (4.9-6.1)	
35-49	58188169	26.8 (26.4-27.1)	16.6 (15.9-17.2)	15.2 (14.2-16.1)	14.4 (13.3-15.5)	
50-64	59629883	24.8 (24.3-25.2)	25.3 (24.3-26.2)	26.4 (24.8-28)	33.4 (31.8-35.0)	
65+	48712209	16.8 (16.4-17.1)	33.6 (32.6-34.6)	36.9 (35.3-38.5)	42.3 (40.2-44.4)	
Sex						<.001
Male	114071243	49.2 (48.8-49.6)	46.0 (45.0-47.0)	40.6 (39.0-42.1)	42.8 (40.7-45.0)	
Female	122480484	50.8 (50.4-51.2)	54.0 (53.0-55.0)	59.4 (57.9-61.0)	57.2 (55.0-59.3)	
Race/ethnicity						<.001
NH white	152886446	63.6 (63.0-64.1)	69.1 (68.1-70.1)	69.2 (67.3-71.1)	67.9 (66.0-69.7)	
NH black	27618505	11.8 (11.4-12.1)	11.0 (10.2-11.9)	10.5 (9.3-11.7)	12.6 (11.4-13.9)	
NH NAA	1255177	0.5 (0.4-0.5)	0.5 (0.4-0.6)	0.8 (0.6-1.1)	1.1 (0.7-1.6)	
NH NHOPI	806711	0.4 (0.3-0.4)	0.2 (0.1-0.3)	0.2 (0.1-0.3)	0.3 (0.1-0.5)	
NH Asian	13041090	6.2 (5.9-6.5)	2.9 (2.5-3.3)	2.7 (1.9-3.6)	1.7 (1.2-2.2)	
NH 2+ races	4048452	1.5 (1.4-1.6)	2.1 (1.9-2.4)	3.2 (2.6-3.8)	2.9 (2.2-3.6)	
Hispanic	36895346	16 (15.6-16.5)	14.1 (13.5-14.7)	13.3 (11.8-14.9)	13.5 (11.9-15.1)	
Marital status						<.001
Married	122886850	54.0 (53.6-54.5)	46.0 (44.9-47.1)	40.6 (38.9-42.3)	39.5 (37.3-41.6)	
Not married	113664877	46.0 (45.5-46.4)	54.0 (52.9-55.1)	59.4 (57.7-61.1)	60.5 (58.4-62.7)	
Education						<.001
Not high school graduate	28068126	10.3 (10.1-10.6)	15.0 (14.3-15.7)	19.6 (18.3-20.8)	26.5 (24.7-28.4)	
High school graduate	58951843	23.3 (22.9-23.6)	30.5 (29.6-31.4)	33.2 (31.6-34.7)	32.6 (30.5-34.7)	
Some college/associate degree	73531775	31.0 (30.6-31.3)	32.3 (31.3-33.2)	32.0 (30.4-33.6)	28.3 (26.0-30.5)	
College graduate	75999983	35.4 (34.9-35.9)	22.3 (21.2-23.3)	15.2 (14.2-16.3)	12.6 (11.3-13.9)	

© 2023 Blais RK et al. *JAMA Network Open.*

Employment						<.001
Employed	147832875	68.5 (68.1-68.9)	47.2 (46.3-48.2)	32.1 (30.6-33.5)	16.2 (14.9-17.6)	
Unemployed	88718852	31.5 (31.1-31.9)	52.8 (51.8-53.7)	67.9 (66.5-69.4)	83.8 (82.4-85.1)	
Family income						<.001
<$ 20,000	37355287	13.2 (12.9-13.5)	22.0 (21.1-22.9)	28.1 (26.8-29.4)	38.2 (36.5-39.9)	
$20,000-49,999	68314946	27.3 (26.8-27.7)	34.3 (33.5-35.1)	37.6 (35.9-39.2)	36.0 (34.2-37.9)	
$50,000-74,999	37775373	16.4 (16.2-16.7)	14.9 (14.1-15.6)	13.9 (12.5-15.2)	12.0 (10.3-13.8)	
$75,000+	93106121	43.1 (42.6-43.6)	28.8 (27.8-29.9)	20.5 (19.2-21.7)	13.7 (12.3-15.1)	
Insurance coverage						<.001
Yes	213458643	89.8 (89.6-90.0)	91.3 (90.7-91.8)	92.2 (91.5-93.0)	94.1 (93.2-95.0)	
No	23093084	10.2 (10.0-10.4)	8.7 (8.2-9.3)	7.8 (7.0-8.5)	5.9 (5.0-6.8)	
Urbanization						<.001
Metro	202568465	86.5 (86.1-86.9)	83.1 (82.3-83.9)	81.0 (79.5-82.6)	79.7 (77.9-81.6)	
Not metro	33983262	13.5 (13.1-13.9)	16.9 (16.1-17.7)	19.0 (17.4-20.5)	20.3 (18.4-22.1)	
Ever Smoked						<.001
Yes	144010155	59.5 (59.1-59.9)	65.3 (64.3-66.2)	67.7 (66.1-69.2)	68.1 (66-70.1)	
No	92541572	40.5 (40.1-40.9)	34.7 (33.8-35.7)	32.3 (30.8-33.9)	31.9 (29.9-34)	
Alcohol Dependence						<.001
No	228906823	97.1 (96.9-97.2)	96.0 (95.6-96.3)	94.6 (94.1-95.2)	95.3 (94.5-96.2)	
Yes	7644904	2.9 (2.8-3.1)	4.0 (3.7-4.4)	5.4 (4.8-5.9)	4.7 (3.8-5.5)	
Illicit Drug Dependence						<.001
No	231366279	98.3 (98.2-98.4)	96.5 (96.2-96.7)	94.9 (94.1-95.6)	95.2 (94.4-96)	
Yes	5185448	1.7 (1.6-1.8)	3.5 (3.3-3.8)	5.1 (4.4-5.9)	4.8 (4-5.6)	
Health Status						<.001
Excellent	49274970	24.0 (23.7-24.3)	10.9 (10.4-11.5)	5.2 (4.6-5.9)	2.9 (2.1-3.6)	
Very good	85901677	39.8 (39.4-40.2)	27.8 (26.8-28.8)	18.5 (17.3-19.7)	8.7 (7.5-9.8)	
Good	69047678	28.1 (27.7-28.4)	36.5 (35.6-37.4)	33.8 (32.4-35.3)	24.2 (22.4-26.0)	
Fair/poor	32327403	8.2 (7.9-8.4)	24.8 (23.9-25.8)	42.4 (40.8-44.0)	64.3 (62.5-66.0)	
Number of ED visits						<.001
0	176854241	78.3 (78.0-78.6)	66.4 (65.4-67.4)	56.6 (54.7-58.5)	46.3 (44.6-48.0)	
1	33709050	13.0 (12.7-13.2)	18.4 (17.5-19.3)	20.3 (18.9-21.8)	21.8 (20.3-23.3)	
2	16769146	6.2 (6.0-6.4)	8.9 (8.3-9.6)	12.2 (11.0-13.4)	14.3 (13.0-15.6)	
3+	9219290	2.6 (2.4-2.7)	6.2 (5.9-6.6)	10.9 (10.0-11.8)	17.6 (15.9-19.2)	
Number of chronic conditions						<.001
0	136742666	63.0 (62.6-63.4)	42.8 (41.9-43.7)	32.8 (31.7-33.9)	23.7 (21.9-25.4)	
1	63881044	25.7 (25.4-26.0)	32.0 (31.2-32.8)	33.2 (31.5-34.9)	31.3 (29.4-33.2)	
2+	35928016	11.3 (11.0-11.6)	25.2 (24.2-26.1)	34.0 (32.6-35.4)	45.0 (42.8-47.2)	
Depressive episode						<.001
Yes	17464535	4.6 (4.5-4.7)	14.5 (13.9-15.0)	23.3 (22.1-24.5)	26.0 (24.1-27.9)	
No	219087192	95.4 (95.3-95.5)	85.5 (85.0-86.1)	76.7 (75.5-77.9)	74.0 (72.1-75.9)	

© 2023 Blais RK et al. *JAMA Network Open.*

Abbreviations: NH, non-Hispanic; ED, emergency department; CI, confidence interval; NAA, Native American/Alaska Native; NHOPI, Native Hawaiian/Other Pacific Islander

© 2023 Blais RK et al. *JAMA Network Open.*

eTable 3. Multiple Imputation Sensitivity Analysis for Adjusted[a] Multinomial Logistic Regression Model of Suicide-Related Outcomes by Disability and Veteran Status (N = 241 675)

	**Suicidal ideation only** (vs. no suicide-related outcome)			**Suicide planning without attempt** (vs. no suicide-related outcome)			**Suicide attempt** (vs. no suicide-related outcome)		
**Characteristic**	**AOR (95% CI)**	***p***	**AME (95% CI)**	**AOR (95% CI)**	***p***	**AME (95% CI)**	**AOR (95% CI)**	***p***	**AME (95% CI)**
**Number of disabling limitations, by veteran status**									
**Veteran**									
No limitations[b]	1.00	NA	NA	1.00	NA	NA	1.00	NA	NA
1 limitation	**1.51 (1.02-2.23)**	**0.040**	**0.01 (0-0.03)**	0.79 (0.44-1.43)	0.423	0 (-0.01-0)	**2.72 (1.56-4.76)**	**<.001**	**0.01 (0-0.01)**
2 limitations	**2.76 (1.75-4.36)**	**<.001**	**0.04 (0.02-0.06)**	1.98 (0.94-4.19)	0.074	0.01 (0-0.02)	1.51 (0.72-3.16)	0.280	0 (0-0.01)
≥3 limitations	**2.25 (1.39-3.67)**	**0.001**	**0.03 (0.01-0.05)**	**2.87 (1.63-5.04)**	**<.001**	**0.01 (0-0.02)**	**3.60 (1.49-8.70)**	**0.004**	**0.01 (0-0.02)**
**Nonveteran**									
No limitations[b]	1.00	NA	NA	1.00	NA	NA	1.00	NA	NA
1 limitation	2.01 (1.79-2.25)	<.001	0.02 (0.02-0.02)	2.49 (2.10-2.94)	<.001	0.01 (0-0.01)	1.99 (1.69-2.35)	<.001	0 (0-0)
2 limitations	2.38 (2.20-2.81)	<.001	0.03 (0.02-0.03)	3.23 (2.67-3.91)	<.001	0.01 (0.01-0.01)	2.94 (2.43-3.54)	<.001	0.01 (0-0.01)
≥3 limitations	2.93 (2.54-3.38)	<.001	0.04 (0.03-0.04)	3.94 (2.88-5.39)	<.001	0.01 (0.01-0.02)	6.21 (4.88-7.90)	<.001	0.02 (0.01-0.02)
**No limitations**									
Nonveteran[b]	1.00	NA	NA	1.00	NA	NA	1.00	NA	NA
Veteran	1.19 (0.93-1.51)	0.164	0 (0-0.01)	1.77 (1.22-2.57)	0.003	0 (0-0.01)	1.04 (0.63-1.72)	0.873	0 (0-0)
**1 limitation**									
Nonveteran[b]	1.00	NA	NA	1.00	NA	NA	1.00	NA	NA
Veteran	0.89	0.512	0	0.56	0.028	0	1.43	0.180	0

© 2023 Blais RK et al. *JAMA Network Open.*

	(0.63-1.26)		(-0.02-0.01)	(0.34-0.94)		(-0.01-0)	(0.85-2.40)		(0-0.01)
**2 limitations**									
Nonveteran[b]	1.00	NA	NA	1.00	NA	NA	1.00	NA	NA
Veteran	1.37 (0.91-2.06)	0.129	0.02 (0-0.04)	1.08 (0.53-2.22)	0.827	0 (-0.01-0.01)	0.54 (0.29-0.98)	0.041	0 (-0.01-0)
**≥3 limitations**									
Nonveteran[b]	1.00	NA	NA	1.00	NA	NA	1.00	NA	NA
Veteran	0.91 (0.57-1.46)	0.702	0 (-0.02-0.02)	1.29 (0.80-2.06)	0.294	0 (0-0.01)	0.60 (0.28-1.29)	0.193	-0.01 (-0.02-0)

Note. Bolded associations are significant at $p \leq .05$.
a. Adjusted for: age, sex, race/ethnicity, marital status, education, employment status, household income, insurance status, urbanization, smoking status, alcohol dependence, illicit drug dependence, health status, number of emergency department visits, number of comorbidities, depressive episode, and year of survey. b. Reference group.
Abbreviations: AOR, Adjusted odds ratio; CI, confidence interval; AME, average marginal effect; NA, not applicable.

© 2023 Blais RK et al. *JAMA Network Open.*

eTable 4. Multiple Imputation Sensitivity Analysis For Adjusted[a] Multinomial Logistic Regression Models of Suicide-Related Outcomes Among Those With Suicidal Ideation

	**Suicide planning without attempt** (vs. suicidal ideation only[b])					**Suicide attempt** (vs. suicidal ideation only[b])				
**Characteristic**	**AOR**	**95% CI**	***p***	**AME**	**95% CI**	**AOR**	**95% CI**	***p***	**AME**	**95% CI**
**Number of disabling limitations, by veteran status**										
**Veteran**										
No limitations[b]	1.00	NA	NA	1.00	NA	1.00	NA	NA	1.00	NA
1 limitation	**0.46**	**0.23-0.92**	**0.027**	**-0.12**	-0.21- -0.02	1.56	0.75-3.23	0.231	0.05	-0.04-0.15
2 limitations	0.74	0.32-1.73	0.490	-0.05	-0.18-0.09	0.49	0.21-1.14	0.098	-0.06	-0.15-0.02
≥3 limitations	1.25	0.60-2.61	0.545	0.05	-0.09-0.18	1.59	0.58-4.40	0.372	0.06	-0.05-0.18
**Nonveteran**										
No limitations[b]	1.00	NA	NA	1.00	NA	1.00	NA	NA	1.00	NA
1 limitation	**1.33**	**1.09-1.62**	**0.004**	**0.05**	0.02-0.08	1.05	0.88-1.24	0.608	0	-0.02-0.03
2 limitations	**1.39**	**1.13-1.72**	**0.002**	**0.06**	0.02-0.09	**1.24**	**1.03-1.49**	**0.024**	**0.03**	**0-0.05**
≥3 limitations	**1.42**	**1.03-1.95**	**0.035**	**0.06**	0.01-0.11	**2.02**	**1.54-2.66**	**<.001**	**0.09**	**0.05-0.13**
**No limitations**										
Nonveteran[b]	**1.00**	**NA**	**NA**	**1.00**	NA	1.00	NA	NA	1.00	NA
Veteran	**1.57**	**1.01-2.44**	**0.046**	**0.08**	0-0.15	0.90	0.52-1.58	0.720	-0.02	-0.08-0.04
**1 limitation**										
Nonveteran[b]	**1.00**	**NA**	**NA**	**1.00**	NA	1.00	NA	NA	1.00	NA
Veteran	**0.55**	**0.32-0.94**	**0.028**	**-0.09**	-0.16- -0.02	1.35	0.76-2.37	0.305	0.03	-0.05-0.12
**2 limitations**										
Nonveteran[b]	1.00	NA	NA	1.00	NA	1.00	NA	NA	1.00	NA
Veteran	0.84	0.41-1.70	0.625	-0.03	-0.15-0.10	0.36	0.18-0.71	0.003	-0.11	-0.17- -0.04
**≥3 limitations**										
Nonveteran[b]	1.00	NA	NA	1.00	NA	1.00	NA	NA	1.00	NA
Veteran	1.39	0.74-2.62	0.306	0.06	-0.06-0.18	0.71	0.32-1.60	0.406	-0.04	-0.14-0.05

© 2023 Blais RK et al. *JAMA Network Open.*

Note. Bolded associations are significant at $p \leq .05$.
a. Adjusted for: age, sex, race/ethnicity, marital status, education, employment status, household income, insurance status, urbanization, smoking status, alcohol dependence, illicit drug dependence, health status, number of emergency department visits, number of comorbidities, depressive episode, and year of survey. b. Reference group.
Abbreviations: AOR, Adjusted odds ration; CI, confidence interval; AME, average marginal effect; NA, not applicable.

© 2023 Blais RK et al. *JAMA Network Open.*
