# Supplementary material for: Suicide Ideation, Plans, and Attempts Among Military Veterans vs Nonveterans With Disability
Source: JAMA Netw Open. 2023 Oct 13;6(10):e2337679. doi: 10.1001/jamanetworkopen.2023.37679 (PMC10576218; doi:10.1001/jamanetworkopen.2023.37679)
Supplement: Supplement 2. — Data Sharing Statement [file jamanetwopen-e2337679-s002.pdf]

Data Sharing Statement

Blais. Suicide Ideation, Plans, and Attempts Among Military Veterans vs Nonveterans With Disability. *JAMA Netw Open*. Published October 13, 2023.
doi:10.1001/jamanetworkopen.2023.37679

Data
Data available: Yes
Data types: Other (please specify)
Additional Information: The dataset is a publically available dataset. We can make all syntax and output available by request.
How to access data: https://www.samhsa.gov/data/release/2020-national-survey-drug-use-and-health-nsduh-releases
When available: With publication

Supporting Documents
Document types: None

Additional Information
Who can access the data: All code will be made available by request.
Types of analyses: raw data
Mechanisms of data availability: This is a publically available dataset.
